# Case Report: Non-typhoidal *Salmonella* infections transmitted by reptiles and amphibians

**DOI:** 10.3389/fped.2023.1278910

**Published:** 2023-11-23

**Authors:** Benoît Bernar, Nina Gande, Aline Bernar, Thomas Müller, Jörn Schönlaub

**Affiliations:** ^1^ Pediatrics I, Department of Pediatrics, Medical University of Innsbruck, Innsbruck, Austria; ^2^ Pediatrics II, Department of Pediatrics, Medical University of Innsbruck, Innsbruck, Austria

**Keywords:** *Salmonella*, sepsis, newborn, reptiles, *Salmonella enterica*

## Abstract

Non-typhoidal *Salmonella* infections (NTSI) can cause bacterial diarrhea, mostly leading to self-limiting gastroenteritis. However, in at-risk populations, NTSI can have severe complications. As transmission is most commonly foodborne, infection is rare in the breast- or bottle-fed very young. Another route is increasingly implicated, however, in newborns and infants especially: Contact with reptiles and amphibians. We describe infection with *Salmonella enterica subsp. enterica* ser. Monschaui (*S.* Monschaui), transmitted from bearded dragons, in a three-week-old boy. The boy initially appeared well, on the next morning deterioration was dramatic, with tachypnea, tachycardia, and mottled skin. Gram-negative sepsis was documented on day 2. His case prompted a review of published instances of reptile- and amphibian-associated salmonellosis (RAAS), summarized here. Association of *S.* Monschaui infection with exposure to reptiles and amphibians prompted inquiry into household pets. The parents had kept bearded dragons (Pogona sp.), the last of which died two weeks before the patient was born; confirmation of colonization with *S.* Monschaui was thus precluded. Among 63 reports (−5,000 cases) of RAAS or *S.* Monschaui, 62 appeared between 1995 and 2022, 10 were single case reports, and 53 were original articles with −5,000 cases; vectors included turtles, frogs, lizards, and snakes. RAAS is not a new phenomenon, but its incidence recently has risen due to the increased popularity of reptiles and amphibians as non-traditional pets. These animals can carry *Salmonella sp.* and transmit it to humans, posing a risk particularly to infants and other vulnerable persons. Risk mitigation requires that those bringing such pets into the home be informed of dangers associated with reptile and amphibian contact; that those selling reptiles and amphibians be mandated to inform customers comprehensively may be in order.

## Introduction

Non-typhoidal *Salmonella* infections (NTSI) are a common cause of bacterial diarrhea (1, 2). In most cases, NTSI lead to a self-limiting gastroenteritis; however, in populations such as the immunocompromised, the elderly, and infants, NTSI can have severe complications. Most transmission is foodborne, particularly via undercooked or un-cooked meat and eggs. This is why, in general, the infection rate increases after the first year of life (3). Another route is increasingly implicated in newborns and infants: Contact with reptiles and amphibians.

## Case report

### Initial presentation

The parents of a boy aged 3 wk, born at 38 wk gestation (birth weight 3,360 g/50th percentile), after an uneventful pregnancy, brought him to our pediatric emergency department because he had become irritable. He had been weaned from the breast one week before, receiving formula milk by bottle. Fever, diarrhea, and vomiting had not occurred. The parents denied illness in the family, exposure to complementary foods, or contact with other infants.

The boy appeared generally well on initial examination, which found nothing specific. While waiting for test results, the parents noticed apnea and cyanosis, which improved after prompt stimulation. Repeated examination again found no abnormalities. A first blood draw, analyzed by point-of-care-testing (HORIBA EU Group companies, Microsemi CRP) revealed as sole finding of note a leukopenia (3,200/µl, expected 5,000–21,000/µl), the C-reactive protein (CRP) value was unremarkable at 0.5 mg/dl (expected 0–0.5 mg/dl). Leukopenia and the parents' report of apnea with cyanosis prompted hospital admission during the late evening. Although the night passed uneventfully, on the next morning deterioration was dramatic, with tachypnea, tachycardia, and mottled skin.

### Diagnostic assessment

**Figure 1:**
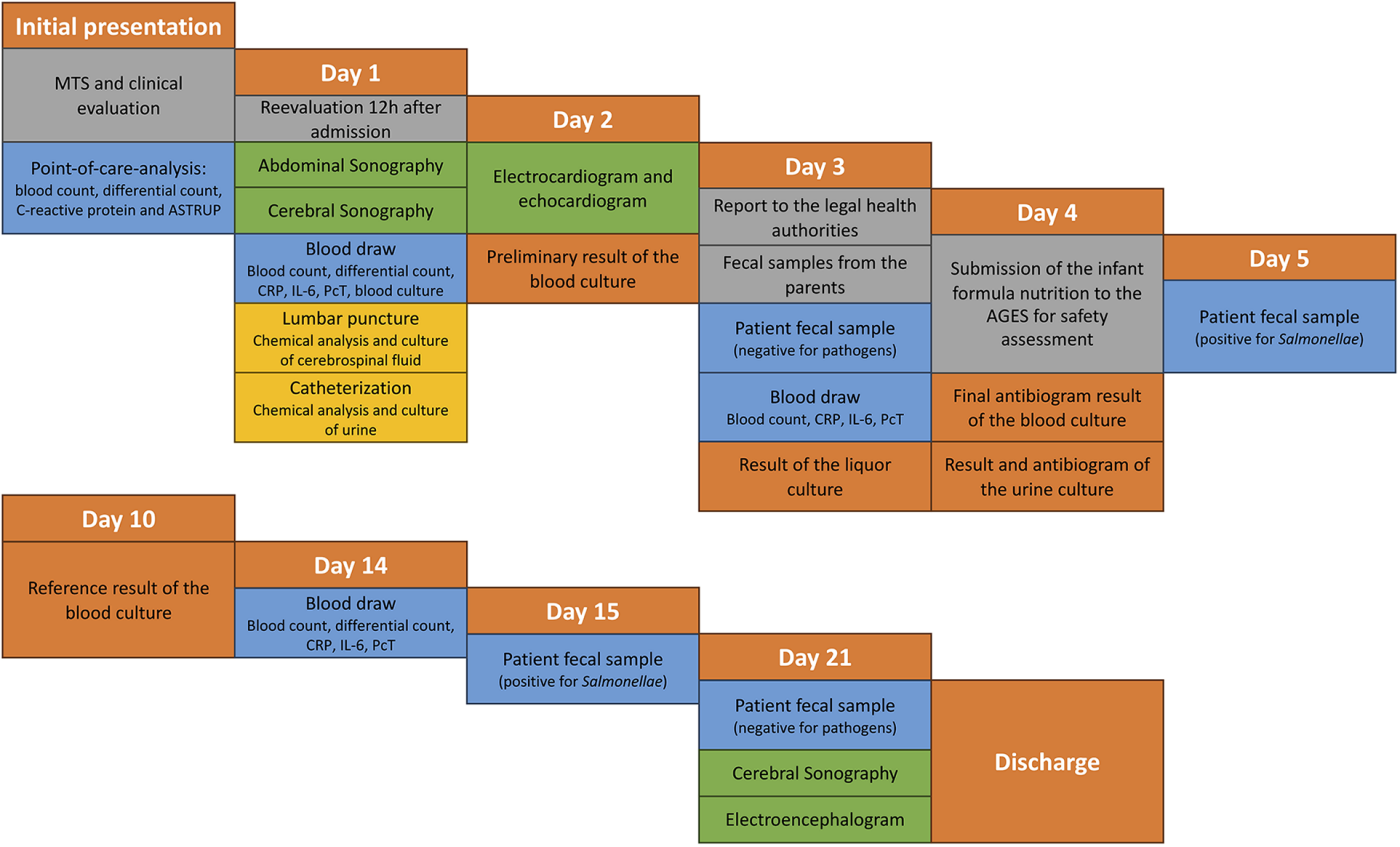
Diagnostic procedures. WBC (White blood cell count), CRP (C-reactive protein), IL-6 (interleukin-6), PcT (procalcitonin), PLT (platelets), HR (heart rate), BR (breathing rate), RR (blood pressure), EEG (electroencephalogram), AGES (Austrian Health and Food Safety Agency), MTS (Manchester Triage System).

**Figure 2:**
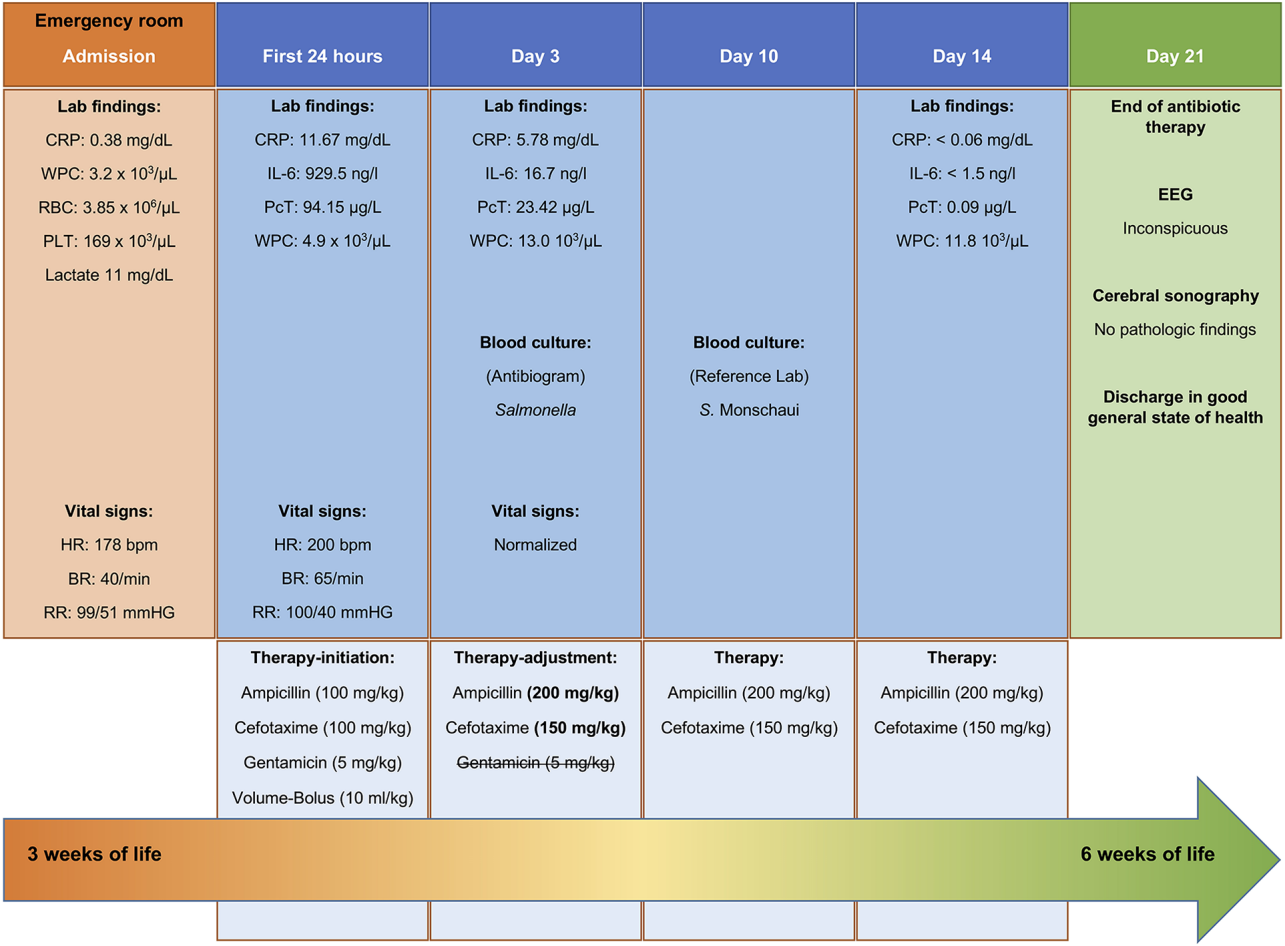
Principial lab results and therapy-timeline. WBC (White blood cell count), CRP (C-reactive protein) expected 0–0, 5 mg/dl, IL-6 (interleukin-6) expected 0–50 ng/L, PcT (procalcitonin) expected 0–0.5 µg/L, PLT (platelets), HR (heart rate), BR (breathing rate), RR (blood pressure), EEG (electroencephalogram).

On the morning following admission, in suspicion of a late-onset sepsis, we commenced a comprehensive diagnostic assessment (Figure 1) and established venous access. Blood studies revealed biomarker values indicative of inflammation (Figure 2), and the blood counts were as follows: 4,900/µl for white blood cells, 4.9 T/L for erythrocytes, and 244 G/L for thrombocytes.

Urine was obtained via catheterization, with negative results for leukocyte esterase, nitrite, bilirubin, and ketones. The pH was 5, protein was measured at 250 mg/L, erythrocytes at 10/µl, and glucose levels were within the normal range. A lumbar puncture was performed, revealing clear cerebrospinal fluid with the following chemical results: glucose 59 mg/dl, protein 393 mg/L, L-lactate 12.9 mg/dl, 3 leucocytes/µl, and no other types of cells.

### Therapeutic decisions

We suspected a late-onset sepsis, urosepsis or meningitis. An empiric antibiotic therapy was initiated immediately following the diagnostic procedures (see Figure 2). The antibiotic selection was based on our patient's age and the potential spectrum of microorganisms, leading to a triple therapy. We decided to use cefotaxime as broad-spectrum antibiotic and to close potential gaps with ampicillin (Gram-positive spectrum, especially *Enterococci*) and gentamicin (*Listeria monocytogenes*).

Gram-negative sepsis was documented on hospital day 2 when rod-shaped bacteria, subsequently identified as *Salmonella sp.*, were detected in cultured blood. Time to positivity was 8.42 h and the sample was subsequently sent to the Austrian *Salmonella* reference laboratory for further analysis and serotyping (Austrian Health and Food Safety Agency, IMED Graz).

Legal requirements prompted us to report the infection to the health authorities. Simultaneously, fecal samples were collected from the parents and our patient. Pathogenic microorganisms, particularly *Salmonellae*, were not detected in the fecal samples from the parents. However, *Salmonellae* were subsequently detected in our patient's stool (see Figure 1), with no other pathogenic microorganisms identified. Serotyping of stool samples was not performed.

The antibiogram, conducted in Innsbruck, indicated that the microorganism was sensitive to all three antibiotics. This prompted us to discontinue gentamicin (to reduce the risk of toxicity) and optimize cefuroxime and ampicillin (to prevent meningitis and its potential higher risk of a fatale outcome). However, the cerebrospinal fluid culture was sterile. In addition to the *Salmonella* identified in the blood culture, *E. coli*, sensitive to all three antibiotics, was moderately cultured from the urine sample (with over 10^4^cfu/ml). However, the urine macroscopically appeared clear, had no conspicuous smell, and there was no presence of leukocytes. Therefore, despite the moderate detection of *E. coli* in the catheter urine meeting the European criteria (4, 5) for a urinary tract infection, it was not further addressed by us due to the lack of additional therapeutic implications (sensitive to the antibiotic therapy already in use) and hence, in contrast to the significantly more severe *Salmonella* sepsis, we considered it as an incidental finding. In accordance with the recommendations from “UpToDate”, “AWMF” and based on specific scientific studies (6, 7), we decided to continue the dual antibiotic therapy for a total duration of three weeks.

The patient responded well to treatment and recovered rapidly and showed only mild indications of gastrointestinal distress. Abdominal sonography found only intraluminal bowel gas in possibly excessive quantities. Cerebral sonography and electroencephalography at the end of antibiotic therapy found no abnormalities; neurologic status and overall condition were unremarkable. *Salmonella* sp. was no longer detectable in feces on the day of discharge or in a follow-up specimen 2 week later.

### Route of infection

The route of infection was obscure for some time. *Salmonella sp*. was found neither in parental feces nor in the formula milk given the patient. The formula milk was analyzed before by the Austrian Health and Food Safety Agency. The parents denied feeding the patient meat or dairy products. They had no pets.

The solution came only with the result from the reference laboratory. The sample was serotyped using the White-Kauffmann-Le Minor Schema and revealed a *Salmonella enterica subsp. enterica* ser. Monschaui (35:m, t:-). *S.* Monschaui is a rare infective agent and is associated with exposure to reptiles and amphibians (8). Informed of this, the parents stated that, although they had no pets when their son fell ill, the father before had earlier kept goliath spiders, snakes, and most recently two bearded dragons (*Pogona* sp). Both lizards had died unexpectedly, the second 2 week before the patient was born, and their bodies had been discarded (making them not available for further analyses). Thereafter the father had disinfected all accessible surfaces of the apartment and sterilized the terrarium and its decorations with boiling water. Given that the parents had previously owned bearded dragons, the rarity of the microorganism, which is primarily associated with reptiles, the fact that the infant formula was pathogen-free, and the negative stool samples from the parents implicated, on the balance of probabilities, fomitic transmission from a colonized surface to the infant.

### Outcome

Due to the good overall condition, further check-ups could be carried out by the pediatrician in the outpatient setting after discharge. At age 18 months, the patient was hospitalized for assessment of gastroesophageal reflux disease. He was in excellent general health, with age-appropriate developmental and neurologic status. His parents stated that *Salmonella sp*. was intermittently detected in fecal samples (further serotyping was not performed) during his first postnatal year but not thereafter: After the first year of life, several consecutive stool samples had remained free from *Salmonella*. The patient is now 4yrs old, active, in an excellent general state of health and with an age-appropriate neurological development.

## Literature review and discussion

No instance of a newborn infant infected by *Salmonella enterica subsp. enterica* ser. Monschaui transmitted from bearded dragons was discovered in a computerized search of PubMed (https://pubmed.ncbi.nlm.nih.gov/) and Google Scholar (https://scholar.google.com/) using the terms “reptile- and amphibian-associated salmonellosis”, “reptile associated salmonellosis”, “amphibian associated salmonellosis”, “reptile-associated salmonellosis” and “*Salmonella* Monschaui”.

Newborns infected by *S.* Oranienburg (9) and *S.* Apapa (10) transmitted by indirect or direct domestic contact with bearded dragons are reported, as are instances of infections with other *Salmonellae*, transmitted from other reptiles such as snakes, in newborns or young infants (3, 8). Contact with reptiles still appears rarely recognized and/or underestimated as a mode of infection in newborn sepsis. It is essential that parents and caregivers be aware of this risk.

*Salmonella* sp. are Gram-negative, rod-shaped, facultative anaerobic bacteria, family *Enterobacteriaceae*, commonly cause zoonoses (1). *Salmonellae* survive well outside host organisms, even on stainless steel surfaces for at least 1 month (11). At present >2,600 serovars of *Salmonella* are known, among them *S.* Monschaui; most are forms of the species *Salmonella enterica* (12, 13). *S.* Monschaui, isolated from an infected wound of a soldier who stepped on a mine in Monschau in Germany in 1945, was first described in 1946 (14).

Whilst salmonellosis typically manifests as a transitory gastroenteritis with diarrhea and dehydration, in populations such as newborns and infants it can have severe complications, including sepsis (15). Sepsis incidence shows age-related peaks in infants aged <1year and in the elderly, with males more commonly affected in both (16). Mortality of *Salmonella* sepsis is high at 24.3% (16) and is highest among the elderly (33.5%–36.5% in those aged >80years) (16). Infants aged <1year also have a high in-hospital mortality rate, up to 3.9%, and even higher for severe sepsis (17.6%) and septic shock (39.5%) (16). Timely initiation of antimicrobial therapy is critical, as a delay of >3 h is associated with higher rates of organ failure and increased mortality (17). *Salmonella* meningitis in newborns is of particular concern. Whilst *Salmonella* is identified as causative in only 1% of cases of bacterial meningitis in newborns and infants (18), associated mortality and morbidity are considerable. In developing countries, mortality rates have been as high as 75% (19), and even in developed countries only about one-third of survivors have a favorable outcome (6).

### Path of transmission and reptile-associated transmission rate

In both adults and children, non-typhoidal *Salmonellae* (NTS) transmission is commonly feco-oral via contaminated food (12). However, −11% of all NTS infections are associated with animal contact (20), with transmission via claw scratches or bites (21). Between 2009 and 2018, 1.465 reported cases of RAAS in the United State led to 306 hospitalizations (22, 23).

NTS can infect a wide range of animals, including pigs, poultry, cattle, and reptiles. In 1963, turtle-associated human salmonellosis was first described in the United States (24). Since then, the number of reported RAAS has continuously increased. Incidence data, however, are sparse and depend on the outbreak rate, which varies from year to year; between 2009 and 2018 yearly multistate outbreaks varied in number between 0 in 2013 and 6 in 2015 (22). In 1975, the United States in response banned the sale of small turtles. Sweden repealed a similar law in 1996 and in 1997 the number of *Salmonella* infections tripled (25). In the United States, information on reptile-associated outbreaks of disease is regularly published (24, 26). Up to 90% of reptiles carry *Salmonella* (23, 27).

*Salmonella* isolates were found more often in reptiles bought in pet shops (88.9%) than in wild-caught animals (58.8%) (27). In Europe RAAS cases are irregularly published and incidence rates are unclear. The Robert Koch-Institut (RKI) reported in 2013 that RAAS increased in number from 50 in 1997–99 to 154 in 2006–08 and to 178 in 2009–11 (28). In the Netherlands yearly case numbers increased from 1985 to 2014, with estimated incidence ranging from 11 cases in 1988 to 93 cases in 2013; however, the increase was principally in adults (29). The percentage of RAAS among all instances of salmonellosis increased from −10% in 2006 to −35% in 2011 (28). In southwest England 48 among 175 children aged <5year between 2010 and 2013 had contact with reptiles (30). A recent Italian report of 2 studies found that RAAS represented from 5.7% to 11.7% of instances of sporadic salmonellosis in individuals <21year (31).

### Modern pets: reptiles and amphibians

Whilst RAAS is not truly an emergent disorder, the incidence of infection seems to be rising as reptile ownership has become more frequent. Reptiles and amphibians are nontraditional pets (NTP), unlike dogs and cats. Between 1996 and 2017 –81% (197/243) of disease outbreaks associated with NTP were attributed to *Salmonella* infections and among them −25% (62/243) were reptile-transmitted (15). These 243 outbreaks comprised 9.798 infected persons (15). Between 2012 and 2014 166 patients with RAAS were identified, among whom 71 reported contact with bearded dragons; *S.* Monschaui was not reported (32).

In 2022 nearly 11.000.000 reptiles (number of households unknown) were estimated to be held as pets in Europe (in Austria 125.000) (33); nearly 6.000.000 households in the United States hold reptiles (absolute number of reptiles unknown) (34). Between 1990 and 1999, 1.3 million reptiles covered by the Convention on International Trade in Endangered Species of Wild Fauna and Flora were imported into the European Union (35). At the beginning of the 1990s yearly import numbers approximated 60.000 reptiles, reaching −250.000 at the end of the decade (35). According to the Tierärztliche Grenzkontrollstelle Hessen (Veterinary Border Checkpoint Hesse) at Frankfurt (Main) airport, nearly 800.000 reptiles and amphibians were imported into the European Union by air through Frankfurt in 2008 (36).

European records confirm that not only have reptile imports increased, the incidence of RAAS has also risen. Data from The European Surveillance System (TESSy) indicate that infections with reptile-associated *Salmonella* serovars have been on the rise in children aged <3year (28). According to the RKI, *Salmonella* infections in infants have decreased overall since 2001. However, infections with rare serovars have increased in the same population (28). Since 2006, the incidence of RAAS has risen to triple the average rate between 1997 and 2005 (28). In children aged <2year the proportion of reptile-associated salmonellosis within salmonellosis overall has also tripled between 2006 and 2011 (28). Regarding specific serovars, RKI data reveal that *S.* Monschaui infection increased in incidence between 2007 and 2010 in children aged <3year, although it remains relatively rare with <20 cases yearly (28).

RAAS can affect anyone, but members of certain groups are particularly vulnerable to severe clinical courses. These high-risk groups include those aged <5year, those aged >65year, the immunocompromised, and the pregnant (15).

The Centers for Disease Control and Prevention (CDC) reported 8 multistate outbreaks of *Salmonella* infections linked to turtles from 2011 to 2013, with 13 multistate RAAS outbreaks between 2014 and 2018 (20). The average age of those affected was 4year; 31% were infants (37). The CDC thereupon advised that those aged <5year, those with weakened immune systems, and those aged >65year should avoid handling or touching amphibians, reptiles, or their habitats (38). The RKI and the Austrian Agentur für Gesundheit und Ernährungssicherheit (Austrian Health and Food Safety Agency/AGES) have given similar advice. The RKI states that whilst reptiles can be kept if species-appropriate as domestic animals, they should not be pets for children (28). The AGES advises that households with infants aged <1year should not harbor reptiles under any circumstances (39).

Of note is that the father of our patient was not aware of the risk of salmonellosis associated with reptiles, despite owning several NTPs for many years, including snakes and Goliath spiders. This emphasizes that vendors of such pets must be required to inform purchasers of disease risks and to warn that the pets should remain terrarium confined.

## Limitations

Fomite transmission from a colonized surface to the infant is the most likely route of transmission; however, we couldn't obtain conclusive evidence for this probable theory since the previously deceased bearded dragons had already been disposed and were, thus, unavailable for further examination.

The results of the subsequent fecal cultures were not available to us after discharge, which is why we can only provide anamnestic data regarding the further course during the first year of life.

## Conclusion and perspective

RAAS is well-known. Its increasing incidence has been linked to increasing popularity of NTPs. NTSI usually result in self-limiting gastroenteritis, but people in certain groups may experience severe complications, such as sepsis and meningitis, which carry high morbidity and mortality. Consequently, prompt diagnosis is crucial, and initiation of empirical antibiotic therapy should not be delayed.

RAAS poses a significant and often underestimated risk, especially for infants. That parents (or prospective parents) be aware of this potential danger is essential. NTP vendors should be mandated to provide comprehensive risk information to customers to ensure public awareness and safety.

## Data Availability

The original contributions presented in the study are included in the article/Supplementary Material, further inquiries can be directed to the corresponding author.
